# Some Account of the Prevailing Epidemic in the Northwest, Variously Designated, but Usually Popularly Denominated “Spotted Fever”

**Published:** 1864-06

**Authors:** J. Adams Allen

**Affiliations:** Prof. Prin. and Prac. Med. and Clin. Med., Rush Med. College; Formerly Prof. etc., in the Indiana Med. College; Late Prof., etc., in the Med. Dep’t of the University of Michigan; Ex-President of the State Med. Society of Michigan, etc.


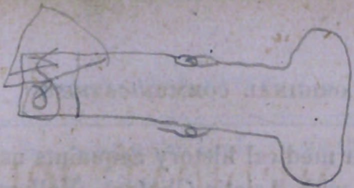

CHICAGO

MEDICAL JOURNAL.

VOL. XXI.] JUNE, 1864. [No. 6.

### ORIGINAL COMMUNICATIONS.

### SOME ACCOUNT OF THE PREVAILING EPIDEMIC IN THE NORTH-WEST, VARIOUSLY DESIGNATED, BUT USUALLY POPULARLY DENOMINATED "SPOTTED FEVER."

A Paper Read Before the Illinois State Medical Society, May, 1864.

By J. ADAMS ALLEN, M. D., LL. D.,

*Prof. Prin. and Prac. Med. and Clin. Med., Rush Med. College; Formerly Prof. etc., in the Indiana Med. College; Late Prof., etc., in the Med. Dep't of the University of Michigan; Ex-President of the State Med. Society of Michigan, etc.*

The peculiar severity and remarkable prevalence of the epidemic popularly called Spotted Fever, and the great anxiety which manifestly prevails, to glean from every quarter information with regard to its nature, and especially its treatment, must be my apology for trenching upon the limited time of the Society by a brief paper upon the subject.

The writer claims as large an experience in this form of disease as has probably fallen to the lot of any living member of the profession, as it has happened to have been his fortune to pass through as wide a spread and devastating epidemic of it, as any with which medical history acquaints us.* Limited in range, compared with Asiatic Cholera, Yellow Fever, or the Plague, nevertheless, by its intensity and mortality, in the particular districts which it attacks, the writer does not hesitate in saying that it is vastly more to be dreaded in any community than either of the forms of disease mentioned.

In the present paper the writer must premise the regret that fire, which is no respecter of persons, destroying his residence and its contents, in the spring of 1851, spared not his memoranda of this terrible epidemic which devastated many parts of the State of Michigan during the years 1847, 8, and 9. A record of numerous cases, in their onset, progress, and *post-mortem*, was thus unfortunately destroyed. The impression left upon the writer's mind, deepened as it has been by frequent observations since that time, is too profound to depend upon any manuscript aid. Not less than five States of the North-West have recently been visited by this mortal disorder. A very little light is better than none in a dark place, and it is not hazarding too much to say that no man as yet so well understands the "Spotted Fever," as to be incommoded by the slightest additional information on the subject.

Of the Spotted Fever very little is found in the text-books, and our own observation is, that of the various monographs on the subject, but few, if any, reflect the personal experience of their writers.† Even the name is a subject of dispute—*stat nominis umbra.*

The diagnosis is comparatively clear to any one with even slight experience—unfortunately the reliable therapeutics puzzle and confound the most expert. Most remarkable as an epidemic, yet it often occurs sporadically, and in the latter case the diagnosis usually is—*congestion* of this, that, or the

*The number of cases personally observed by the writer, was not less than a thousand, and probably many more than that number.

† Several monographs have appeared on the subject, wherein the writers speak *boldly*, although they confess to have seen not more than a half dozen cases.

other organ, more frequently the brain. As though the term *congestion* explained anything!

In observing the phenomena presented, we may premise that they simulate the toxicological—we can scarcely resist the belief that they are caused by a specific *materies morbi*, which the popular opinion readily conjectures to be a contagious principle, yet by careful observation we become convinced that this *materies morbi* must have been generated within the system by the agency of external causes. The causes without—the poison generated within. According to the writer's observation, it is more likely to occur in winters with a variable temperature—where a few days of intense cold are rapidly followed by days of thaw, mud, and rain. Neither uniformly cold nor warm weather are so likely to engender it. But other influences unquestionably co-operate. It does not seem always confined to a particular season. We have seen cases in the wet basements of Chicago, and they have, according to the best authority, occurred in military camps in damp localities, irrespective of, or connected with "malarious" influences. Here and there, wherever the "grand army" of the Union has been dispersed, it has manifested itself, oftentimes vaguely denominated Cerebro-Spinal Meningitis, or "Congestive Fever," according as the reporter was, or was not, familiar with the peculiarities of the disease.

In its incursion it does not appear to select those debilitated by previous disease, in preference to the robust. It selects no particular age or sex, neither are any exempt. Nor does it seem to be influenced materially by localities—those upon the highlands, and those upon the lowlands—the rich and the poor are quite impartially seized. And yet there is a feature of causation which is generally to be noticed as common—the variable hygrometric and thermometric condition of the atmosphere. This is a point worthy of consideration in its physiological relations, and gives a glimpse of the real nature of the disease.

The Attack.—The modes of attack are various, and yet all coincide in reprosenting a potent influence upon the nervous system. More commonly this is manifested by distinct *rigor* gradually passing to general coldness, absence of pulse in the extremities, and coma, very like that of pernicious remittent, and this, it may be, without marked local symptoms. At other times *pain* is the prominent symptom—pain more frequently in the occipital or nuchal region, but very often in other parts of the head, in the shoulders, trunk, hips, or extremities. This pain is usually sharply circumscribed, but this is not invariably the case. It becomes more and more intense, until the patient grows delirious, apparently from its severity, and then ultimates in coma, after which, the disease takes the same course as when ushered in by a chill. Or it may speedily be replaced by the rigor. The *suddenness of the attack, and rapid progress to grave symptoms* are very characteristic. The delirium which ensues upon the pain, or which supervenes upon the coma, is of the most violent character, with incessant jactitation and often vociferous and incoherent cries.

The Eruption.—Sometimes at the very outset, but usually not until the lapse of several hours—from three to twenty-four—there occurs a peculiar discoloration or eruption upon the surface, but this is exceedingly variable in its form. Sometimes it consists in simple petechial points, and again of dark discolorations of large surfaces, like ecchymosis, or rather the subcutaneous extravasation of purpura. More frequently there is no elevation of the cutis, but occasionally there are elevations of the epidermis like bullæ, with thin, dark, sanious contents. Sometimes it approximates the mulberry rash of typhus, and again it is said by some of my correspondents to look like the eruption of measles. I have never seen it take the latter appearance. The form of the eruption, although from its frequency is derived the popular designation of the disease, is not diagnostic. Mainly the evidences presented by it are those of a diffluent disintegrated state of the blood. Yet I have fancied the prognosis was modified, as in many eruptive diseases, by the brightness or lividity of the eruption. *The eruption or discoloration is not invariably present.*

INNERVATION.—Among the most remarkable symptoms may be mentioned persistent tonic contraction of the muscles of the back of the neck, sometimes deepening into opisthotones; convulsions, paralysis of the extremities, particularly of the arms, and also of the optic and auditory nerves—the auditory oftenest,—remarkable soreness of the surface and joints to touch or motion.

There may be intolerance of light and sound, but oftener the special nerves involved are paralyzed. While there is the most excruciating hyper-æsthesia of the general surface, the fingers may be placed on the cornea with no flinching by the patient. Rarely the muscles of articulation and deglutition are paralyzed. Hemiplegia is not infrequent, or the paralysis may be confined to a single extremity. Strabismus, unequal size of the pupils, twitching of the facial muscles, etc., etc., are incidental symptoms.

THE PULSE, at the outset, is small, thready, tremulous, irregular, soft, and speedily lost at the wrist. On the occurrence of reaction, or remission, it increases in fullness and force. About always during the primary stage, it is slow, but during reaction it runs up to 120 or 150.

THE SKIN is, in most cases, dry as well as cold, but in perhaps twenty-five per cent. it is moist. In all instances, it is variable at different hours of the day.

THE TONGUE is more or less enlarged and flabby, with indentations of the teeth upon its edges. It is pale and moist, with a coating of a pale ash or white color, passing to yellow or brown, but it does not present distinctive symptoms.

NAUSEA AND VOMITING are occasional, but not constant symptoms; the matters thrown up being the normal contents of the stomach, or, in bad cases, decomposed blood, bile, mucus, etc.

THE BOWELS are variable. Some cases are natural, in others, there is obstinate constipation, and in others still, severe diarrhœa, with discharges of grumous and offensive matters.

THE RESPIRATION is ordinarily laborious and irregular, sighing and perhaps stertorous, or hissing through the clenched teeth; more frequent than normal while consciousness continues, but becoming slower with advancing coma.

THE URINE is generally scanty, smoky, albuminous, depositing a plentiful sediment, intermixed with ropy mucus and disorganized blood corpuscles. In some instances I have known it abundant and of low specific gravity.

THE SECOND STAGE.—The first stage may terminate in death in a few hours, or, after an interval of from six to twenty-four hours, there may occur a marked remission of the symptoms —a very deceptive remission—leading both the physician and patient to believe the difficulty pretty much over. Supervening upon the remission rapidly, or it may be upon a gradual awakening from the comatose state, there will be intense febrile excitement, great heat of the surface, rapidity and strong tension of the pulse, arterial throbbings, flushed face, turgidity of the conjunctiva, with extreme thirst, etc. This is liable to be attended with the highest grade of delirium, and frantic muscular jactitation, requiring several attendants, perhaps, to keep the patient in bed.

The delirium may, after a while, merge again in coma, apparently from utter exhaustion, or it may gradually moderate in violence and pass into the low muttering character, with subsultus, etc., the patient supine, with the extremities fixed, and painful to touch or motion; or in some cases turned rather to the prone position, with, in either case, the posterior muscles of the neck fixed rigidly, drawing the head strongly backward. Occasionally it is drawn laterally backwards, but is always in a state of rigid immobility.

In this stage the bowels are inactive, and the urine either scanty or wholly retained. The skin is hot, but not infrequently reeking with an offensive perspiration. Irregular or partial sweats are common. Convulsions, paralysis, pain or other evidences of central nervous disorder are largely aggravated.

The patient may die in this stage, or

LATER, may pass on with irregular febrile phenomena, into the low, lingering typhoid stage, stretching over even forty or sixty days, before the establishment of a tedious convalescence. The low fever which so often supervenes upon severe attacks of the Asiatic Cholera much resembles it.

From the outset there will be presented intermingled symptoms, according to the involvement of particular organs in secondary lesion. The lungs are especially liable to severe or even fatal disorder. So much so indeed as, by the gravity of its manifestations almost entirely to mask the original disease. Dr. Condie, in a note to the American edition of Watson's Practice, includes a description of this form under the head of Typhoid Pneumonia, but it is unnecessary to say that the disease described is but the one now under notice, with secondary pulmonary complication.

Ulceration of the throat, the writer has observed as a complication in several cases—the destruction of tissue proceeding to an alarming extent. Erysipelas is the most frequent and dangerous of complications. It is easily kindled upon the slightest abrasion of the surface, and often, without such a nidus, attacks the face and neck, extending to the scalp, or more rarely seizing the extremities. This erysipelatous affection is apt to be exceedingly destructive to the part attacked, giving rise to foul sloughing sores, rapidly wearing out the patient by exhaustion. Some of our friends who have observed this frequent concurrence of erysipelas, have been so struck by it as to be led to believe the disease essentially an erysipelatous inflammation of the Cerebro-Spinal tissues, as also of those organs secondarily involved.

The abdominal viscera sometimes experience the full force of the morbific influence, but in greater part are comparatively little disturbed. Epistaxis, hæmatorrhœa, hæmatemesis, and hæmaturia are occasionally present to a dangerous extent.

SEQUELA.—Among the sequela we note deafness, blindness, partial or complete paralysis of motion, tuberculosis, albuminuria. Albumen is about invariably present in the urine of the tardily convalescent, or ultimately fatal cases of long continuance. Marasmus in both children and adults, and phthisis in the latter, are especially common. The constitution seems broken down, and perhaps years elapse before the surviving patient recovers his original vigor.

Post Mortem.*—With regard to *post mortem* appearances, there has been much apparent discrepancy of description. The explanation of this discrepancy is found easily in the fact that death may result in any stage of the disease. It thus becomes quite difficult, at times, to discriminate the incidental complication from the primary original affection.

If the patient die in the first stage, within a few hours from the attack, there may be very few signs of local change. The blood will be found settling to dependent portions, and with a loose, gory, diffluent clot. There will be found stasis of blood in the capillaries of the Cerebro-Spinal meninges, particularly of the pia-mater and dura-mater, rarely of the arachnoid. The superficial vessels of the convolutions will be distended, and those upon the walls of the venticles likewise full, presenting here and there spots like ecchymosis, though more florid in hue. Sections of the cerebral substance will show a similar condition of the blood vessels, as manifested by large red points on cutting across them. The medulla oblongata, spinal meninges, and cord, at the upper part, will exhibit similar appearances. The stasis of semi-dissolved blood is so considerable that both the gray and white tissues gain a pinkish tinge.

More or less circumscribed softening is found, usually involving the cortical substance of the cerebrum, the inferior surface of the cerebellum, and the floor of the lateral ventricle. In fine, a lessening of the firmness and density of the whole mass is evident on careful inspection. Effusion of discolored serum into the cavities is present, now and then, to a large amount.

* The post mortem appearances here described, are those fully confirmed by the writer's own dissections.

If the case has run on to high reaction, all the results of inflammation may be present. In this case the arachnoid is apt to be thickened, and with loss of its transparency. In the exuded matters are found pus and lymph corpuscles, or the exuded lymph forms a quasi membrane, loose, creamy, and readily broken down, upon the surface, or along the course, of the large vessels. The greatest quantity is at the base of the brain, and always at the optic commissure. It is also stated to be found on the corpora quadrigemina, the medulla oblongata, and around the third pair of nerves, where they penetrate the arachnoid membrane. Wherever the exudation takes place it is likely to be stained by the dissolved hæmatin.

In the spinal region, the morbid changes affect mainly the meninges, the substance showing little modification of structure—the most observable being softening. The exudation, discoloration, and softening are most noticeable about the roots of the cervical nerves. The thoracic and abdominal lesions present nothing distinctive. Where the lungs become involved, there are the ordinary evidences of stasis of blood and effusion, or where the later stage is reached, the usual results of inflammation. The same remark may be made of the intestinal tube.

Here rests the case, so far as my own examinations have extended, and so far as those made by others, which have fallen under my notice, have been concerned. To my own mind the morbid anatomy thus far developed, is altogether unsatisfactory. The advances of modern Physiology indicate a still further exploration of the cadaver, which I confess not to have made, and am at this time only consoled in not having made, by the concurrent failure of other inquirers on this subject, and by the still more cogent reflection that probably if the examination had been made it would have proved scarcely more than negative.* But on this point hereafter.

*At the meeting of the State Society, before which this paper was read, it was stated that certain recent dissections showed commencing fatty degeneration of the kidneys. In so far this confirms the writer's present views.

It is proper to remark, that whilst in about every case there is on *post mortem* some visual evidence of local disease of the cerebro-spinal region, nevertheless, in very many, this evidence is very slight, and in some it is inappreciable. The disease is capable of killing the patient speedily or slowly, and yet leaving behind no trace of its footsteps. Here, as elsewhere, the facts teach that the immediate molecular changes, upon which life and death depend, are intrinsically beyond the sphere of our aided or unaided observation. We necessarily infer their existence, precisely as we infer the reactions of a chemical mixture, by observation of results, and of things which we can see, and handle, and weigh.

Necræmial Cases.—In common with many other potent influences which suddenly strike down the powers of life—the cause, or causes of this terrible disease may produce, at once, necræmial symptoms, so profound that particular manifestation of the special "proximate cause" will not take place. The diagnosis here is really not of much, if of any, importance. Malignant typhus, small pox, scarlatina, rubeola, diphtheria, Asiatic cholera, erysipelas, pernicious remittent, uræmia, etc., are severally capable of producing the same condition. In this case, an overwhelming cause acts especially upon the cerebro-spinal centre, and secondarily upon other organs. The blood from the beginning is poisoned, and scarce living, and death may ensue from this poisoning, or later, from the local changes in the brain and cord, or in some instances, from involvement of other vital organs. Probably all observable lesions are truly secondary.

Diagnosis.—The only forms of disease with which this is liable to be confounded are, pernicious remittent, ("congestive") fever and typhus. Nevertheless, as there is no limit to human absurdity, one medical journalist claims that all these noted cases are but malignant scarlatina! Such absurdity of speculation exhibits itself in too clear a light to need effort at controversy. Yet it may be observed that in the progress of pernicious remittent and malignant typhus, cerebro-spinal changes often occur, which render the diagnosis impossible; and this may not be deemed unfortunate, because at the same time it is unnecessary. Since enlightened medical men have ceased to regard diseases as entities, and no longer rely upon routine treatment, the diagnosis of names has yielded place to the search for causes and conditions, and treatment has been placed upon a broader and more certain basis.

Those who carefully notice the peculiar disturbances of the nervous system and muscular apparatus in this affection, can not fail to see that they are exceedingly rare in the so-called congestive fever of malarious districts. There is no periodicity about it. It can not be merely a severer form of that fever for it occurs in districts indiscriminately, where that fever is known and where it is not. It occurs at a season of the year, mainly, when notoriously, pernicious remittent loses its virulence, and indeed, is scarcely ever present, save in cases which have been especially liable to attacks of periodical disease, and whose constitutions have been broken down by previous illness.

*Briefly*, the paralysis of special sensation and voluntary motion; the hyperæsthesia of the surface and joints, or more remarkably, of circumscribed localities; the opisthotonos and convulsions; the petechiæ and vibices; the absence of periodicity, notwithstanding marked remissions; the locality and times of occurrence; the sequela; these and many other particulars, to be seen by the seeing eye, distinguish the disease from "Congestive Fever," with no difficulty or uncertainty.

The influence of quinia is wonderfully diagnostic. In pernicious remittent it is prompt, powerful, and certain—it is *the* remedy, with all others ignored. In the disease under review, it is scarcely noticeable in effect, and wholly unreliable, nay, comparatively worthless as a remedy. The "*Congestive Fever*," bows beneath its potent influence at once—the "*Spotted Fever*" scorns its impression.

Practically, the *physiognomy* of the disease is as diverse as the lineaments of strangers.

Nearly the same features separate it from "maculated typhus." It is not a severer form of typhus disease, because it is rarely developed under those known circumstances which develop the latter. The nervous manifestations at the outset are wholly diverse; the mode of attack and progress of the cases are dissimilar, save that in some cases a low fever follows the secondary lesions; but well marked, and indeed some of the severest, cases are arrested, and convalescence occurs, without any such secondary fever.

The eruption, or rather extravasation of dissolved blood beneath the skin, is wholly unlike the maculæ of typhus, or the roseolar spots of typhoid. Such extravasations do not constitute the peculiar eruption of typh disease. Typh fevers are the creation of *ochlesis*, of bad ventilation, filth, and improper food. This clearly depends on meteorological influences, entirely apart from the accumulation of human beings in crowded places; independent of decomposing organic matter; independent of confined air. It attacks equally all ages, from the infant to the veteran—the out-door laborer and the lady in the parlor.

Presenting some symptoms in common with typh diseases, its history and physiognomy are as diverse from them as "bilious remittent" from "yellow fever."

Its diagnosis only becomes difficult from them in that necræmial condition, before alluded to, when these and a vast number of other malignant diseases are merged in common manifestations, by the overwhelming nature of the causes producing them.

Prognosis.—The proportionate mortality in the early periods of this, as of other pestilential epidemics is something frightful to contemplate; but there always remains this well-established fact that under about any respectable, or even so-called active, treatment, the fatality steadily diminishes with time, and the later cases are quite controllable. Its primary mortality in proportion to the number seized, is unsurpassed even by Asiatic cholera.

Therapeutics.—It would be a happy circumstance if the treatment were as satisfactorily determined as even the character of the disease. Unhappily this is not the case—but much can be done, and many lives thereby saved, which neglected would have been lost. This is a truth which charlatans and pretenders are ready to take advantage of, and all ears are speedily stunned by the praises of particular remedies and methods which they go around braying about. "Specifics" for it are as plentiful in every city, village, and hamlet as applicants for offices after a Presidential election—but, alas, they fail miserably in the trial.

Under the writer's own observation has come treatment which ought to make all classes of created things—from angels to jackasses—weep. We have seen men try to bring on a crisis—the *death-crisis*—which we have elsewhere exposed, by bloodletting. Others attempt the same thing by emetics and mercurial purges. Steaming is perhaps the most popular plan of producing exhaustion of the feeble remaining powers of life. We have seen long trains of wagons returning through *Michigan mud* from the hemlock woods, a score or more of miles away, laden with the precious branches, (Birnam's wood coming to Dunsinane,) greeted as Oriental pilgrims coming from Mecca.

I have known fifty grains of morphia given within a dozen hours to a boy of fifteen, to relieve him from the terrible pain and suffering, with no avail, save that death followed.

Incalculable quantities of brandy and quinine, of capsicum and carbonate of ammonia, have been poured into the stomachs of the comatose; and there is no measurement of the amount of turpentine and other irritants, which have been thrown up, *per rectum.* The surface has been parboiled, roasted, cauterized. What have we not seen done?

There is no limit to human credulity—there is no bound to human assumption. The most trivial remedies have been ushered into notice, and temporary repute, under the loosest of professional hypotheses—fortunate only in that they have replaced destructive methods.

Flimsy chemical analogies, mixed with apochryphal ferments, have floated sundry inert substances into use, which luckily convert the sin of commission into the less odious sin of omission. Satisfactory treatment remains to be discovered. There are no specifics—there are no antidotes. Yet experience points to some things which seem to have done something—perhaps, *post hoc ergo propter hoc*, after all.

And, *first*, we rank external stimulants—rubefacients and artificial heat. Whatever will powerfully stimulate the surface, especially along the spine and the extremities. This not from any vague idea of internal "pressure," or "congestion," to be overcome by "determining to the surface," but for promoting tissue changes in this most accessible of the excretory organs, and thus awakening energy of the circulation, and immediately excretion of effete matters, and renewal of blood The rubefacient becomes thus a general stimulant. With capsicum and mustard, ammonia and terebinthinates, pungent oils and friction, the means are abundant enough, and the practitioner can take his choice. But, let it be observed, to be useful the action of the surface must be awakened, and not merely irritated. Persistency, and yet caution not to destroy the tissue, must be the cardinal points in view. Direct heat from its ultimate sedative impression, is a more questionable remedy. The hot water or vapor bath speedily exhausts—still, when the surface is dry and rigid, these may be employed for a short time only. The alcoholic vapor bath is better.

A potent stimulant is the application of ice for a few moments and then the alternation of hot epithems—this alternation to be frequently repeated. The intense local pain, so often present in the primary stage, may occasionally be controlled by full doses of morphia internally, but usually the amount required is so excessive as to prove dangerous of itself. Under these circumstances the hypodermic injection will be found of greater advantage. But from the puncture there is always danger of erysipelas. The same remark applies to vesicants—primarily of excellent service, secondarily there is danger of sloughing sores. The slightest abrasion of the surface is to be looked upon with fear.

Internally stimulants and restoratives, boldly, but with due regard to their ultimate effect. Thus the alcoholic stimulant of whatever sort, when acting at all, is quite disposed to produce the sedative or anæsthetic effect, checking tissue metamorphosis, hence the disappointment so often experienced on its administration during the cold stage, or in necræmial cases. If given, it is better combined with arterial stimulants—capsicum, piperin, monarda, etc. Etherial preparations, ammonia, etc., answers well in some cases.

In the writer's experience the *Tincture of Cantharides* in full, and what to some would seem inordinate, doses has been found the most valuable of internal stimulants. Of this, (a *proved* preparation,) from twenty to forty drops may be given every hour until reaction ensues, or strangury supervenes. The occurrence of strangury, so far from being dreaded, is to be hailed as an omen of the most favorable import. It may be but a coincidence, but it is fortunately an invariable one, that the patient in whom this symptom occurs, from this or any other cause, gets well. This interesting fact, observed more than fifty years ago in the history of this same disease, seems to us to let in a glimpse of light upon the therapeutics. It is not the strangury which cures, but the awakening of the nervous system and of the excretory energy, which this symptom incidentally manifests. The favorable result may be secured without the induction of strangury.

Where there is any appearance of erysipelas, or where there are any evidences of dissolution of the blood, as shown by the deep, livid shade of the spots, or by passive hæmorrhages, the *Muriated Tincture of Iron* should be simultaneously administered. Coinciding with the general effect of the Cantharides, it seems to especially prevent further destruction of the blood and to facilitate its repair. Besides it lessens the liability to injury of the kidneys, and moderates strangury. Ten, twenty, or more, drops of the Tincture of Iron may be given with each dose of the Cantharides, and the mixture well diluted in some convenient vehicle.

Other stimulating diuretics which we have tried have thus far proved less useful in our experience, but we favor further experiment on the point.

When pain is dangerously intense, or convulsions occur, chloroform or ether may be carefully administered by inhalation; and we should very much like to try here, as we have not yet had an opportunity, the Protoxide of Nitrogen, or "laughing gas." Theoretically, and by analogy of its use in other cases, we are inclined to believe it would be found an invaluable remedy. Its energetic stimulating power would probably favor speedy reaction.

We have not noticed any especial advantage from quinia in the first stage, although we have given it in doses varying from a couple of grains to half a dram every hour. The stimulating effect of the small dose is inappreciable; the sedative and diaphoretic effect of the large dose is objectionable. But when the deceptive remission occurs, we have fancied some benefit was gained by several full doses, at intervals of one or two hours—the sedative effect seemed to lessen the severity of the subsequent exacerbation, although we have found that a hundred grains thus given, would not prevent its access.

Please observe, that while the writer thus ignores quinia as a curative agent of cardinal value, as some believe it, in pure cases of Spotted Fever, he is ready to admit the great probability of its superior efficacy in the management of cases modified by the so-called malarious influences. The same remark may be made with regard to the arsenical preparations and other "anti-periodics."

On the occurrence of the remission, or the conclusion of the first period, something appears to be gained by relieving the bowels with a moderately stimulating cathartic, particularly when the cold stage has left behind it decided engorgement of the portal system. By this means the ensuing exacerbation is ameliorated, and the tendency to pass afterwards into a low form of fever is sensibly lessened. We usually, for this purpose, combine calomel with gamboge, rhubarb, and soap, which operates freely but not violently in six or eight hours. Beyond this or some milder cathartic, we have rarely found it useful to employ purgation. The patient can not be cured by attempts to turn him inside out with either emetics or cathartics.

*If during the stage of excitement which now follows, the urine becomes tolerably free and charged with its solid constituents, the patient may be reckoned safe, but if it is suppressed the case is one of extreme danger.*

The practitioner is apt to be deceived by the apparent excitement, and hence to resort to excessive anti-phlogistic measures, but this is a dangerous error· Collapse impends, almost momentarily, and it is indispensable to be on guard. Saline diuretics, very moderate doses of the usual sedatives, and perhaps anodynes may be used; but bleeding, vomiting, and purging are out of the question. Ablutions with cold water, or the tepid bath, or showering the head, we have found in this case even more useful than in the similar stage of remittent. *When in doubt*, our own experience teaches us to say, *stimulate* even here. At all events hold up the great powers of life by careful restoratives and abundant fluid nutriment. Destructive changes will thus be prevented; the liability to collapse lessened, and the tendency to tedious low fever with doubtful final result, abated.

But better do nothing than to do mischief. Discussion of the low fever and the various sequela occasionally met with, ensuing upon the attack, is not within our present purview. The treatment is comparatively clear and simple. Incidental local complications will modify each case, and the patience of even the most skilled practitioner will be severely taxed by the tedious prolongation. But the indications are so palpable and unmistakable that they need not command further notice in this place.

One or two remarks and this already too long extended paper will be brought to a close. The term Cerebro-Spinal Meningitis now generally accepted as designating "Spotted Fever" is objectionable, in that it conveys the idea that the disease is essentially an inflammation of the parts indicated, which is clearly not the fact.

The phenomena indicate the presence of a blood poison, prominently (but not invariably or exclusively,) affecting the cerebro-spinal meninges.

There is reason to believe that this blood-poison is generated within the body itself, by concurrence of external causes influencing especially the physiologically vicarious, yet collaborating organs, the skin and the kidneys.

There is reason to believe that elimination of that poison, and consequent cure, is to be sought among those agents which especially impress these great emunctories.

At all events, mere stimulants or antiphlogistics fail!

